# Argininemia as a cause of severe chronic stunting in a low-resource developing country setting: a case report

**DOI:** 10.1186/s12887-016-0668-9

**Published:** 2016-08-22

**Authors:** Nora King, Romina Alvizures, Pablo García, Ann Wessel, Peter Rohloff

**Affiliations:** 1 Wuqu’ Kawoq | Maya Health Alliance, 2a Calle 5-43 Zona 1, Santiago Sacatepéquez, Guatemala; 2 Facultad de Medicina, Universidad Francisco Marroquín, 6a Avenida 7-55 zona 10, Guatemala City, 01010 Guatemala; 3 Metabolism Program, Boston Children’s Hospital, 300 Longwood Ave, Boston, MA 02115 USA

**Keywords:** Argininemia, Inborn errors of metabolism, Stunting, Child development, Resource-poor medicine

## Abstract

**Background:**

Argininemia is rare inborn error of metabolism which, when untreated, presents in late infancy with growth delay and developmental regression. In developed countries, argininemia is diagnosed early by newborn screening and is treated immediately with a protein-restricted diet. In developing countries, diagnosis may be delayed by the assumption that stunting is related to malnutrition alone.

**Case presentation:**

We describe the diagnosis and treatment of argininemia in a 60-month-old Kaqchikel Maya girl in rural Guatemala. The patient initially presented with severe stunting and developmental regression. The initial diagnosis of argininemia was made by a screening test in dried blood spots and confirmed with urine and serum amino acid profiles. The patient was treated with a low-protein diet using locally available foods, leading to significant improvement in her growth and development.

**Conclusions:**

This case demonstrates that the identification, diagnosis and treatment of IEM in developing countries are increasingly feasible, as well as ethically imperative. Providers working with malnourished children in developing countries should suspect IEM in malnourished children who do not respond to standard therapies.

## Background

Argininemia (OMIM #207800), an autosomal recessive disorder of the urea cycle, is caused by a deficiency in the activity of the hepatic arginase enzyme (ARG1). It is thought to be among the rarest of the urea cycle disorders (UCD), with a global incidence of 1 in 2 million births [1]. Unlike the other UCD, argininemia does not typically present with neonatal or early childhood hyperammonemia. Rather, the disorder emerges between 1 and 4 years of age, characterized by slowing linear growth and failure of regular cognitive development [2, 3]. Episodic hyperammonemia may occur, but usually does not lead to life-threatening acute illness. If untreated, argininemia can cause loss of developmental milestones, spastic diplegia, and severe intellectual disability.

In developed countries, the early diagnosis of non-proximal UCD is accomplished by tandem mass spectrometry (MS/MS) newborn screening [4]. However, in most developing countries, these techniques are unavailable. Additionally, the exceedingly high prevalence of chronic undernutrition, as well as the relative lack of access to pediatricians, may lead to a failure to consider inborn errors of metabolism (IEM) in the differential diagnosis of growth disorders. Here we report on a case of argininemia diagnosed in a 5-year-old female of Kaqchikel Maya ethnicity in rural Guatemala who previously carried a diagnosis of chronic severe malnutrition. Her diagnosis was aided by international partnerships. We discuss the unique challenges of managing argininemia in a rural, under-resourced setting.

## Case presentation

A 60-month-old female presented to our clinic in central Guatemala after developmental delay since late infancy and developmental regression for 1 year. At this time, her weight was 9.0 kg and height was 86.3 cm. According to the World Health Organization growth standards, her height-for-age Z-score was -4.9 and her weight-for-height Z-score was -3.0, consistent with severe wasting and severe stunting [5].

The patient was born at term at home following an uneventful pregnancy. She had never seen a physician, but was evaluated by several community health workers and received presumptive community-based therapy for undernutrition. She is of Kaqchikel Maya descent and was born in an isolated village in Guatemala. Her parents were first cousins; there was no family history of severe childhood illness or death. She exhibited progressive delay in attaining motor milestones; although she sat independently (5 months) and crawled (10 months) in a timely fashion, she did not walk until 2 years. Language and social development were delayed, and she exhibited only vocalizations and gestures without meaningful word acquisition. She never attained bladder or bowel sphincter control. She had no acute severe childhood illnesses, and received all routine vaccinations according to the national schedule. She was exclusively breastfed until 1 year of age. Upon initiation of complementary feeding, she showed a clear aversion to animal protein and vomited on the rare occasions that she consumed it. At 4 years of age, 1 year before presentation to our clinic, the patient ceased walking, adopted a flexed posture of both lower extremities with scissoring and rigidity, and restrictive eating lead to significant weight loss. The patient’s physical exam was notable for severe under-nutrition, and lower extremity hypertonicity and spasticity. When supported in a standing position, she exhibited scissoring and a “tip-toe” posture. Babinski reflexes were upgoing bilaterally. She demonstrated an attentive gaze and social smile, but was nonverbal. Her hair was lighter and coarser than that of family members. Dietary recall revealed extremely low energy consumption (52 kcal/kg/day) with low-normal protein intake (0.93 g/kg/day) exclusively from plant sources. The family followed the advice of community health workers to provide dietary supplements, including iron, vitamin B12 and fortified corn-soy blends, but felt ambivalent about the treatments’ effectiveness.

Blood chemistry showed borderline elevations of lactic acid (3.3 mmol/L, reference range 0.7-2.1 mmol/L) and ammonia (87.9 ug/dL, reference range 18-90 ug/dL). AST (aspartate aminotransferase) and ALT (alanine aminotransferase) were 38 IU/L and 50 IU/L, respectively. TSH (thyroid-stimulating hormone), uric acid, blood urea nitrogen, creatinine, bilirubin, serum electrolytes, and complete hematologic profile were uninformative. A filter paper blood spot was obtained and transported to the United States by courier. Analysis was conducted by Perkin Elmer Genetics laboratory (Bridgeville, PA). The analysis revealed an elevated arginine level of 3.05 mg/dL (reference range <2.00 mg/dL). On a repeat blood spot, arginine was again elevated at 2.04 mg/dl. Subsequently serum and urine samples submitted to the Biochemical Genetics Laboratory at the Mayo Clinic (Rochester, MN) for confirmatory testing. Results were remarkable for serum arginine of 510 nmol/mL (reference range 31 – 132 nmol/mL) and urine orotic acid of 46 mmol/mol of creatinine (reference range undetectable), findings consistent with a biochemical diagnosis of argininemia.

The mainstay of treatment for argininemia is a protein-restricted diet [6]. This presents a substantial challenge in rural Guatemala, where poverty and food availability prevent many families from providing adequate nutrition for healthy children, much less for special needs diets. Additionally, there is no access to protein-free or essential amino acid medical foods or ammonia-scavenging medications. Corn tortillas, the staple of the Maya Guatemalan diet, provide 200 kcal and 5.4 g protein per 100 g [7]. At even fairly nominal levels of consumption, tortillas would easily exceed recommended daily protein restrictions for a UCD [8]. The family expressed concern about the cost of other low-protein, high-calorie foods. In collaboration with the patient’s family, we developed a customized diet based on locally available food stuffs, principally oat or corn porridge and affordable fruits and vegetables. Refined sugar and vegetable oil were used to augment calories, since these were affordable to the family. This diet provided 110 kcal/kg/day and 1.5 g/kg/day of locally available protein. For the first 4 weeks following the patient’s diagnosis, a Kaqchikel Maya-speaking social worker specializing in child nutrition made weekly visits to review dietary intake.

On this nutritional regimen, she gained 3.3 kg in the first 6 months, and her weight-for-height Z-score improved to -0.9. Importantly, the patient regained developmental milestones. Six months following her diagnosis, the patient was able to turn completely while lying, support her torso from a prone position and stand briefly while supporting herself. Before treatment, she was completely immobile. The patient’s family reported her to be increasingly interactive, and at 6 months basic verbal language returned. Dietary adherence was very good and the family quickly learned to differentiate high- and low-protein foods. Several 24-h diet recalls demonstrated an average of 94 kcal/kg/day and 1.4 g/kg/day of intact protein.

## Conclusions

Here we present a case of late diagnosis of argininemia in a severely stunted and wasted Maya child in Guatemala. Diagnostically, this represented a challenge because of the Guatemala’s endemic stunting, particularly among the rural, indigenous population to which our patient belongs. Indeed, rural indigenous Guatemalan children are likely the most stunted population in the world [9]. Because stunting and chronic undernutrition increase susceptibility to infectious disease, severely stunted children commonly present with acute wasting [10]. Furthermore, severely stunted children can manifest significant cognitive delays and late attainment of motor function [11, 12]. For these reasons, initial attempts by community health workers to treat this patient for the common malnutrition syndromes were not unreasonable. However, several features of the patient’s presentation, including the loss of developmental milestones, progressive spastic diplegia, and highly restrictive eating habits with protein avoidance, were inconsistent with primary malnutrition, prompting us to initiate a more thorough medical evaluation. In addition, her stunting was striking even in comparison to cohorts of other poor, Maya children, who averaged 102.2 cm at 5 years [13]. This highlights the need for protocol- and community-based approaches to child malnutrition in developing countries—potentially in the form of “warning signs” checklists—to better train frontline workers on indications for deviating from protocol and seeking a higher level of care.

The treatment for argininemia requires dietary protein restriction while providing adequate nitrogen and energy for optimal growth. We did not have access to essential amino acid medical foods that typically complement a low-protein diet to provide total daily protein intake at or below recommendations for age. In addition, we were unable to monitor plasma amino acids to evaluate dietary adherence, and extreme poverty limited access to higher-quality protein. We instituted a mild protein restriction of 1 to 1.5 g protein/kg, emphasizing complementary vegetable proteins (e.g., beans and corn). This diet was financially acceptable to the patient’s family, and actually increased the child’s pretreatment protein intake to promote catch-up growth, prevent catabolism, and provide essential amino acids.

Confirming the diagnosis of an IEM in a developing country is challenging given the lack of laboratories that conduct newborn screening and esoteric confirmatory testing. Guatemala’s two largest public hospitals, located in the capital, have small in-house screening programs for detecting congenital hypothyroidism, phenylketonuria, congenital adrenal hyperplasia, and galactosemia only [14]. Our experience offers insight into ways to improve diagnosis and management of UCDs in resource-poor settings. Given the stability of filter paper blood samples, we suggest that international processing in a reference laboratory with extended newborn screening capabilities is a rapid, cost-effective mechanism for advancing the diagnostic workup of children in whom metabolic illness is suspected. We acknowledge that confirmatory testing remains problematic and expensive, especially when scarce funds must be allocated to provide the most societal benefit. However, when evaluating severe presentations of long-standing malnutrition in a referral center such as ours, there is an important need to reconsider the clinical presentation and available data and to broaden the differential diagnosis and workup where resources allow. We suggest that global health practitioners working with children with complex medical illnesses should consider building capacity for collecting, transporting, and processing filter paper specimens.

Finally, the diagnosis and management of IEM in developing countries is frequently assumed to be logistically and financially untenable. Indeed, the medical foods and ammonia-scavenging medications typically used in managing UCDs are not available where we work, nor are laboratory tests for monitoring plasma amino acids. However, our patient had a remarkable clinical response to simple nutritional therapy, including careful total protein restriction with global calorie augmentation using economical local foods. Her case underscores the inestimable benefit of case management and nutrition in under-resourced, global health contexts.

### Consent

Verbal informed consent in Kaqchikel Maya was obtained from the patient’s mother and grandmother, her legal guardians. Verbal consent was deemed to be more ethical than written because the guardians do not speak Spanish or English and cannot read or write. This was done in accordance with the medical organization’s Statement on Informed Consent in Minority Groups (available upon request). The interpreter who participated in the consent signed a witness statement agreeing that it was performed in accordance with ethical principles (also available upon request).

## Abbreviations

ALT: Alanine aminotransferase
AST: Aspartate aminotransferase
IEM: Inborn error of metabolism
TSH: Thyroid-stimulating hormone
UCD: Urea cycle disorder
